# The Circulation of Sap in Trees, and the Formation of Maple Sugar

**Published:** 1865-10

**Authors:** 


### THE CIRCULATION OF SAP IN TREES, AND THE FORMAMION OF MAPLE SUGAR.

Trees are made up of fine tubes which extend from the root to the leaf, and it is through these tubes that the circulation of the sap is carried on. If a growing tree is pulled up by the roots, and the roots are placed in a vessel of water containing some colored solution which they will absorb, we can trace the course of this colored solution through the tree by cutting notches into it at successive periods. The coloring matter is always found first in the body of the wood near the root, then in the wood higher up, and so on until it reaches the leaf; then it begins to appear in the inner bark near the leaf, and it passes down through the bark again to the root. This observation shows that the circulation of the sap is up through the wood, and down through the bark.

There has been much speculation in relation to the force that causes the sap of plants to circulate, but it has never been settled by observation and experiment. It is pretty well established that sap circulates in the winter, though less rapidly than in the summer, and less rapidly at that time in deciduous than in evergreen trees.

The solid portions of thoroughly dried wood, and other parts of plants, are composed mainly of water and charcoal. When charcoal is burned, a small portion of ash is left. This ash is the mineral or inorganic portion of the substance of the tree, and consists principally of potash, lime, and flint or silex. That portion which burns is carbon. In burning, the carbon unites with oxygen to form carbonic acid, an invisible gas that floats away in the atmosphere.

The water and the inorganic matters enter the tree through the roots; the carbon enters mostly through the leaves. Carbon forms about one-half of the solid substance of the tree, and water the other half.

Water is composed of two elements, oxygen and hydrogen, in the proportion of eight pounds of oxygen to one of hydrogen. These in entering into a chemical combination with carbon, lose the liquid state of water, and form the various solid substances which make up the body of the tree.

In its course the sap undergoes important transformations. The trunks and leaves of trees are scenes of constant chemical operations, many of them more mysterious than any of the operations of the laboratory. One of these is the decomposition of carbonic acid in the leaf. The affinity of carbon and oxygen is very strong indeed, and there are few forces in nature that can rend these two elements asunder; but the combined action of light and vegetable life is separating them throughout every day in the leaves of all growing plants. Carbonic acid is absorbed from the atmosphere by the leaf, its two elements are torn apart, the oxygen is returned to the air, and the carbon combining chemically with other elements in the sap, is carried to the places where new wood is being formed, and is there deposited in its proper place to help build up the structure of the tree. The symmetrical order in which the carbon is deposited in a try may be seen by looking at a piece of charcoal.

If wood is examined under a powerful microscope, it is found that the tubes through which the sap circulates are formed of minute sacs or cells. The substance of which the walls of these cells are formed is called cellulose. It has been the subject of a great deal of chemical research, and is found to consist of carbon and water, or more strictly, of carbon and the elements of water, oxygen and hydrogen. Cotton and linen are almost pure cellulose. Each atom of cellulose contains twelve atoms of carbon, ten atoms of hydrogen, and ten of oxygen, $C_{12}\ H_{10}\ O_{10}$. Starch, gum, and sugar ali have the same composition, $C_{12}\ H_{10}\ O_{10}$. This is one of the wonders of chemistry, that substances composed of the same elements, combined in the same proportion, should have properties so different as gum, starch, sugar, and cotton or linen fibre. Their different properties must of course result from the varied modes in which the atoms are arranged.

Besides these four substances there is one other constituting a considerable portion of the body of trees, which is also formed of the same elements as the others, but in slightly different proportions. This is lignin. It is an incrustation on the inner surfaces of the cell walls, and its office appears to be to strengthen and stiffen these walls. Its constitution is $C_{12}\ H_8\ O_8$. In this case, as in the others, there are just as many atoms of hydrogen as of oxygen; these two elements enter into the compound in the same proportion to each other as that in which they unite to form water. If a tree or other plant is thoroughly dried so as to expel all of its uncombined water, nine-tenths of the remaining substance consists of the five compounds, cellulose, lignin, starch, gum, and sugar, and all of these are composed of hydrogen and oxygen in the same relative proportion as that in which they exist in water, chemically combined with carbon.

Why it is that the atoms of these substances are so arranged in one part of the plant to form cellulose, and in another to form starch; why it is that they are so arranged in one tree as to form gum, and in another to form sugar, are mysteries which lie beyond the present boundaries of human knowledge.

There is one other organic element, and several inorganic, besides those mentioned, which enter, though in small quantities, into the constitution of plants, but a full discussion of the part which they perform in vegetabl economy would demand an exhaustive treatise on agricultural chemistry and vegetable physiology.—*Am. Drug. Circular.*

